# Supplementary material for: Investigating the Health Effects of 3 Coexisting Tobacco-Related Products Using System Dynamics Population Modeling: An Italian Population Case Study
Source: Front Public Health. 2021 Nov 16;9:700473. doi: 10.3389/fpubh.2021.700473 (PMC8634955; doi:10.3389/fpubh.2021.700473)
Supplement: Supplementary file 4 [file Data_Sheet_4.pdf]

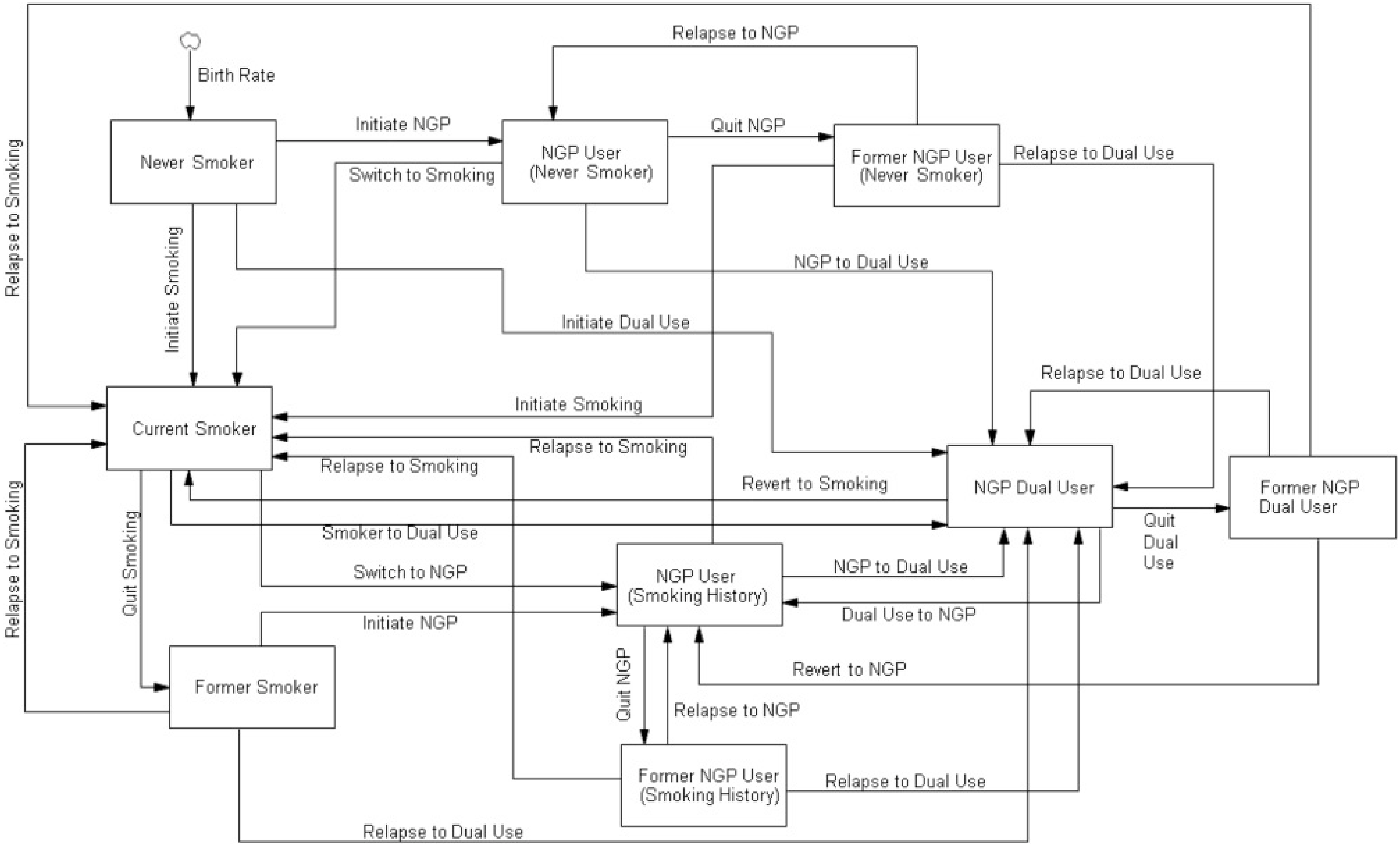
Birth Rate
Never Smoker
Initiate NGP
NGP User (Never Smoker)
Quit NGP
Former NGP User (Never Smoker)
Relapse to NGP
Relapse to Dual Use
Switch to Smoking
Relapse to Smoking
Initiate Smoking
NGP to Dual Use
Initiate Dual Use
Relapse to Dual Use
Current Smoker
Initiate Smoking
Relapse to Smoking
Relapse to Smoking
Revert to Smoking
NGP Dual User
Former NGP Dual User
Smoker to Dual Use
Quit Dual Use
Relapse to Smoking
Quit Smoking
Switch to NGP
NGP User (Smoking History)
NGP to Dual Use
Dual Use to NGP
Initiate NGP
Former Smoker
Revert to NGP
Quit NGP
Relapse to NGP
Former NGP User (Smoking History)
Relapse to Dual Use
Relapse to Dual Use

<table>
<tr><th>Flow Name</th><th>Originating Stock</th><th>Transition Values</th></tr>
<tr><td>Initiate Smoking</td><td>(Never Smoker)</td><td>
<table>
<tr><th>Initiation (%/year)</th><th>Under 14</th><th>14 – 17 Years</th><th>18-19 Years</th><th>20 – 24 Years</th><th>25 – 34 Years</th></tr>
<tr><td>Male</td><td>0.0%</td><td>3.6%</td><td>10.0%</td><td>2.0%</td><td>0.9%</td></tr>
<tr><td>Female</td><td>0.0%</td><td>3.5%</td><td>2.7%</td><td>1.4%</td><td>0.3%</td></tr>
</table>
</td></tr>
<tr><td>Initiate NGP</td><td>(Never Smoker)</td><td rowspan="2">
<table>
<tr><th>Annual Initiation Probability (95% Confidence Intervals)</th><th>E-Cigarettes Only</th><th>Dual User</th></tr>
<tr><td>From Never User</td><td>0.3 (0.2 – 0.3)</td><td>0.1 (0.1 – 0.1)</td></tr>
</table>
</td></tr>
<tr><td>Initiate Dual</td><td>(Never Smoker)</td></tr>
<tr><td>Switch to Smoking</td><td>(NGP User Never Smoker)</td><td>7.1%</td></tr>
<tr><td>NGP to Dual Use</td><td>(NGP User Never Smoker)</td><td>14%</td></tr>
<tr><td>Quit NGP</td><td>(NGP User Never Smoker)</td><td>20.8%</td></tr>
<tr><td>Switch to NGP</td><td>(Current Smoker)</td><td>1.1%</td></tr>
<tr><td>Smoker to Dual Use</td><td>(Current Smoker)</td><td>4.3%</td></tr>
<tr><td>Quit Smoking</td><td>(Current Smoker)</td><td>
<table>
<tr><th></th><th>14-17</th><th>18-19</th><th>20-24</th><th>25-34</th><th>35-44</th><th>45-54</th><th>55-64</th><th>65-74</th><th>75+</th></tr>
<tr><td>Male</td><td>17.1%</td><td>3.5%</td><td>5.3%</td><td>3.1%</td><td>2.8%</td><td>2.8%</td><td>5.1%</td><td>2.9%</td><td>7.4%</td></tr>
<tr><td>Female</td><td>25.0%</td><td>2.8%</td><td>10.2%</td><td>5.0%</td><td>1.8%</td><td>1.3%</td><td>2.9%</td><td>5.2%</td><td>3.9%</td></tr>
</table>
</td></tr>
<tr><td>Revert to Smoking</td><td>(Dual User)</td><td>45.2%</td></tr>
<tr><td>Dual Use to NGP</td><td>(Dual User)</td><td>9.6%</td></tr>
<tr><td>Quit Dual Use</td><td>(Dual User)</td><td>4.3%</td></tr>
<tr><td>NGP to Dual Use</td><td>(NGP Smoking History)</td><td>14%</td></tr>
<tr><td>Relapse to Smoking</td><td>(NGP Smoking History)</td><td>7.1%</td></tr>
<tr><td>Quit NGP</td><td>(NGP Smoking History)</td><td>20.8%</td></tr>
</table>

| | | |
|---|---|---|
| Relapse to Smoking | (Former Smoker) | (see tables below) |
| Initiate NGP | (Former Smoker) | |
| Relapse to Dual | (Former Smoker) | |

| Years Quit | Relapse Prob |
|---|---|
| 1 | 14.80% |
| 2 | 9.92% |
| 3 | 6.65% |
| 4 | 4.46% |
| 5 | 2.99% |
| 6 | 2.00% |
| 7 | 1.34% |
| 8 | 0.90% |
| 9 | 0.60% |
| 10 | 0.40% |
| 11 | 0.27% |
| 12 | 0.18% |
| 13 | 0.12% |
| 14 | 0.08% |
| 15 | 0.05% |
| 16 | 0.04% |
| 17 | 0.02% |
| 18 | 0.02% |
| 19 | 0.01% |
| 20 | 0.00% |

Above values used when NGP not in market.
When NGP available, above values are modified by the respective scaling factor below

| Relapse Destination | Same Product | Alternative Product | Dual Use |
|---|---|---|---|
| Male | 66.6% | 6.7% | 26.7% |
| Female | 74.2% | 16.1% | 9.7% |

<table>
<tr><td>Relapse to NGP</td><td>(Former NGP Never Smoker)</td><td rowspan="3">
<table>
<tr><th>Years Quit</th><th>Relapse Prob</th></tr>
<tr><td>1</td><td>14.80%</td></tr>
<tr><td>2</td><td>9.92%</td></tr>
<tr><td>3</td><td>6.65%</td></tr>
<tr><td>4</td><td>4.46%</td></tr>
<tr><td>5</td><td>2.99%</td></tr>
<tr><td>6</td><td>2.00%</td></tr>
<tr><td>7</td><td>1.34%</td></tr>
<tr><td>8</td><td>0.90%</td></tr>
<tr><td>9</td><td>0.60%</td></tr>
<tr><td>10</td><td>0.40%</td></tr>
<tr><td>11</td><td>0.27%</td></tr>
<tr><td>12</td><td>0.18%</td></tr>
<tr><td>13</td><td>0.12%</td></tr>
<tr><td>14</td><td>0.08%</td></tr>
<tr><td>15</td><td>0.05%</td></tr>
<tr><td>16</td><td>0.04%</td></tr>
<tr><td>17</td><td>0.02%</td></tr>
<tr><td>18</td><td>0.02%</td></tr>
<tr><td>19</td><td>0.01%</td></tr>
<tr><td>20</td><td>0.00%</td></tr>
</table>
Above values used when NGP not in market.<br>When NGP available, above values are modified by the respective scaling factor below
<table>
<tr><th>Relapse Destination</th><th>Same Product</th><th>Alternative Product</th><th>Dual Use</th></tr>
<tr><td>Male</td><td>66.6%</td><td>6.7%</td><td>26.7%</td></tr>
<tr><td>Female</td><td>74.2%</td><td>16.1%</td><td>9.7%</td></tr>
</table>
</td></tr>
<tr><td>Initiate Smoking</td><td>(Former NGP Never Smoker)</td></tr>
<tr><td>Relapse to Dual Use</td><td>(Former NGP Never Smoker)</td></tr>
</table>

<table>
<tr><td>Relapse to NGP</td><td>(Former NGP Smoking History)</td><td rowspan="3">
<table>
<tr><th>Years Quit</th><th>Relapse Prob</th></tr>
<tr><td>1</td><td>14.80%</td></tr>
<tr><td>2</td><td>9.92%</td></tr>
<tr><td>3</td><td>6.65%</td></tr>
<tr><td>4</td><td>4.46%</td></tr>
<tr><td>5</td><td>2.99%</td></tr>
<tr><td>6</td><td>2.00%</td></tr>
<tr><td>7</td><td>1.34%</td></tr>
<tr><td>8</td><td>0.90%</td></tr>
<tr><td>9</td><td>0.60%</td></tr>
<tr><td>10</td><td>0.40%</td></tr>
<tr><td>11</td><td>0.27%</td></tr>
<tr><td>12</td><td>0.18%</td></tr>
<tr><td>13</td><td>0.12%</td></tr>
<tr><td>14</td><td>0.08%</td></tr>
<tr><td>15</td><td>0.05%</td></tr>
<tr><td>16</td><td>0.04%</td></tr>
<tr><td>17</td><td>0.02%</td></tr>
<tr><td>18</td><td>0.02%</td></tr>
<tr><td>19</td><td>0.01%</td></tr>
<tr><td>20</td><td>0.00%</td></tr>
</table>
<br>Above values used when NGP not in market.<br>
When NGP available, above values are modified by the respective scaling factor below
<table>
<tr><th>Relapse Destination</th><th>Same Product</th><th>Alternative Product</th><th>Dual Use</th></tr>
<tr><td>Male</td><td>66.6%</td><td>6.7%</td><td>26.7%</td></tr>
<tr><td>Female</td><td>74.2%</td><td>16.1%</td><td>9.7%</td></tr>
</table>
</td></tr>
<tr><td>Relapse to Dual Use</td><td>(Former NGP Smoking History)</td></tr>
<tr><td>Relapse to Smoking</td><td>(Former NGP Smoking History)</td></tr>
</table>

<table>
<tr><td>Relapse to Smoking</td><td>(Former Dual User)</td><td rowspan="3">
<table>
<tr><th>Years Quit</th><th>Relapse Prob</th></tr>
<tr><td>1</td><td>14.80%</td></tr>
<tr><td>2</td><td>9.92%</td></tr>
<tr><td>3</td><td>6.65%</td></tr>
<tr><td>4</td><td>4.46%</td></tr>
<tr><td>5</td><td>2.99%</td></tr>
<tr><td>6</td><td>2.00%</td></tr>
<tr><td>7</td><td>1.34%</td></tr>
<tr><td>8</td><td>0.90%</td></tr>
<tr><td>9</td><td>0.60%</td></tr>
<tr><td>10</td><td>0.40%</td></tr>
<tr><td>11</td><td>0.27%</td></tr>
<tr><td>12</td><td>0.18%</td></tr>
<tr><td>13</td><td>0.12%</td></tr>
<tr><td>14</td><td>0.08%</td></tr>
<tr><td>15</td><td>0.05%</td></tr>
<tr><td>16</td><td>0.04%</td></tr>
<tr><td>17</td><td>0.02%</td></tr>
<tr><td>18</td><td>0.02%</td></tr>
<tr><td>19</td><td>0.01%</td></tr>
<tr><td>20</td><td>0.00%</td></tr>
</table>
<br>Above values used when NGP not in market.<br>When NGP available, above values are modified by the respective scaling factor below
<table>
<tr><th>Relapse Destination</th><th>Same Product</th><th>Alternative Product</th><th>Dual Use</th></tr>
<tr><td>Male</td><td>66.6%</td><td>6.7%</td><td>26.7%</td></tr>
<tr><td>Female</td><td>74.2%</td><td>16.1%</td><td>9.7%</td></tr>
</table>
</td></tr>
<tr><td>Revert to NGP</td><td>(Former Dual User)</td></tr>
<tr><td>Relapse to Dual Use</td><td>(Former Dual User)</td></tr>
</table>
